# Supplementary material for: Systematic Review and Meta-Analysis of Mass Spectrometry Proteomics Applied to Human Peripheral Fluids to Assess Potential Biomarkers of Schizophrenia
Source: Int J Mol Sci. 2022 Apr 28;23(9):4917. doi: 10.3390/ijms23094917 (PMC9105255; doi:10.3390/ijms23094917)

SUPPLEMENTARY INFORMATION

Systematic Review and Meta-Analysis of Mass Spectrometry Proteomics Applied to Human Peripheral Fluids to Assess Potential Biomarkers of Schizophrenia

João E. Rodrigues [1,2,†], Ana Martinho [1,2,†], Catia Santa [1,2], Nuno Madeira [3,4,5], Manuel Coroa [2,3,4], Vítor Santos [2,3,4], Maria J. Martins [1,2,6], Carlos N. Pato [7], Antonio Macedo [3,4,5,*,‡], Bruno Manadas [1,2,8,*,‡]

[1] CNC—Center for Neuroscience and Cell Biology, University of Coimbra, 3004-504 Coimbra, Portugal;
joao.e.a.rodrigues@gmail.com (J.E.R.); anajmartinho@gmail.com (A.M.); catiajmsanta@gmail.com (C.S.); martins.mjrv@gmail.com (M.J.M.); bmanadas@cnc.uc.pt (B.M.)
[2] CIBB—Centre for Innovative Biomedicine and Biotechnology, University of Coimbra, 3004-504 Coimbra, Portugal; coroaofc@gmail.com (M.C.); vitorsantos74@gmail.com (V.S.)
[3] Faculty of Medicine of the University of Coimbra, University of Coimbra, 3004-504 Coimbra, Portugal;
nunogmadeira@gmail.com
[4] Psychiatry Department, Centro Hospitalar e Universitário de Coimbra, 3004-561 Coimbra, Portugal
[5] CIBIT—Coimbra Institute for Biomedical Imaging and Translational Research, University of Coimbra,
3000-548 Coimbra, Portugal
[6] Medical Services, University of Coimbra, 3004-517 Coimbra, Portugal
[7] Department of Psychiatry and Behavioral Sciences, SUNY Downstate Health Sciences University,
Brooklyn, NY 11203, USA; carlos.pato@downstate.edu
[8] III Institute for Interdisciplinary Research, University of Coimbra (IIIUC), 3030-789 Coimbra, Portugal
* Correspondence: amacedo@ci.uc.pt (A.M.); bmanadas@cnc.uc.pt (B.M.)
† These authors contributed equally to this work.
‡ These authors contributed equally to this work.

Supplementary Tables

**Table S1**	QUADOMICS criteria to evaluate the quality of the -omics research reports included in a systematic review
**Table S2**	Proteins identified in the selected studies as altered in SCZ vs. control. Proteins are described by name, UniProt entry name and accession number, and the type of sample (serum, plasma, PBMCs, whole saliva and saliva). Table presented in a supplementary excel file. (1) Protein identified with NCBI database id. (2) Protein without any information about accession number through UniProt database
**Table S3**	Proteins identified in the selected studies as altered in SCZ vs. BD. Proteins are described by name, UniProt entry name and accession number, and the type of sample (serum, plasma, PBMCs, whole saliva and sweat). Table presented in a supplementary excel file.
**Table S4**	Results summary from the functional enrichment analysis performed in MetaboAnalyst 5.0. Description of the pathways to which the altered proteins belong to, with indication of number of hits per pathway, as well as statistical analysis of the pathway enrichment and the pathway impact. Table presented in a supplementary excel file
**Table S5**	List of the proteins identified as altered in at least 2 different studies with the indication of the overall trend (up- or down-regulation or both) and the indication of the name indicated in the KEGG schemes in Figure 6. Table presented in a supplementary excel file.

TABLE S1. QUADOMICS criteria to evaluate the quality of the -omics research reports included in a systematic review.

**Study Phase : Phase 1, 2, 3, 4**
1. Were selection criteria clearly described?
2. Was the spectrum of patients' representative of patients who will receive the test in practice? *
3. Was the type of sample fully described?
4. Were the procedures and timing of biological sample collection with respect to clinical factors described with enough detail?<br>4.1. Clinical and physiological factors<br>4.2. Diagnostic and treatment procedures.
5. Were handling and pre-analytical procedures reported in sufficient detail and similar for the whole sample? And, if differences in procedures were reported, was their effect on the results assessed?
6. Is the time period between the reference standard and the index test short enough to reasonably guarantee that the target condition did not change between the two tests?
7. Is the reference standard likely to correctly classify the target condition?
8. Did the whole sample or a random selection of the sample receive verification using a reference standard of diagnosis?
9. Did patients receive the same reference standard regardless of the result of the index test?
10. Was the execution of the index test described in sufficient detail to permit replication of the test?
11. Was the execution of the reference standard described in sufficient detail to permit its replication?
12. Were the index test results interpreted without knowledge of the results of the reference standard?
13. Were the reference standard results interpreted without knowledge of the results of the index test?
14. Were the same clinical data available when test results were interpreted as would be available when the test is used in practice? *
15. Were uninterpretable/intermediate test results reported?
16. Is it likely that the presence of overfitting was avoided?

*Applicable only to phase 3 or 4 studies

Supplementary Figures

**Figure S1**	QUADOMICS evaluation of the quality of the proteomics studies included in the systematic review. QUADOMICS criteria defined in **Supplementary Table S1**.
**Figure S2**	Venn diagram of proteins identified as altered in blood samples (serum, plasma, and CSF) in the selected studies of schizophrenia (SCZ) vs. control. The proteins identified as altered in: i) only serum: 111 proteins; ii) only plasma: 45 proteins; iii) PBMCs only: 20 proteins; iv) plasma vs. serum: 20 proteins; v) plasma vs. PBMCs: 1 protein.

Figure S1. QUADOMICS evaluation of the quality of the proteomics studies included in the systematic review. QUADOMICS criteria defined in Supplementary Table S1.

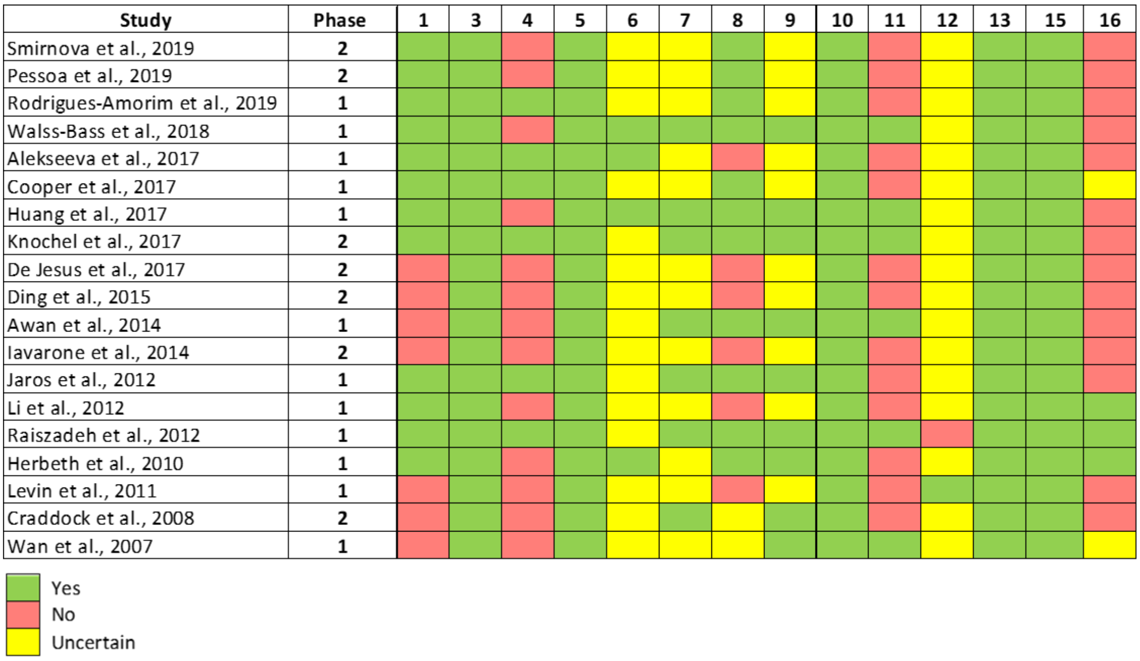

Figure S2. Venn diagram of proteins identified as altered in blood samples (serum, plasma, and CSF) in the selected studies of schizophrenia (SCZ) *vs.* control. The proteins identified as altered in: i) only serum: 111 proteins; ii) only plasma: 45 proteins; iii) PBMCs only: 20 proteins; iv) plasma *vs.* serum: 20 proteins; v) plasma *vs.* PBMCs: 1 protein**.**

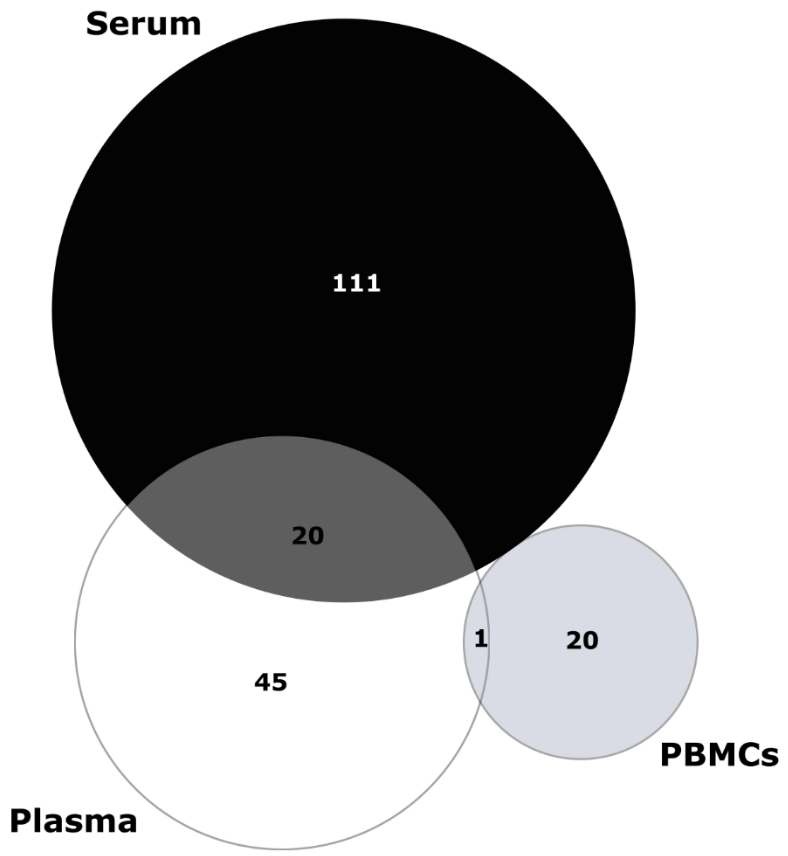

Supplement: Supplementary file 1 [file ijms-23-04917-s001.zip › Supp Info_SCZ Systematic review_IJMS_2022.pdf]
